# Intersection of effort and risk: ethological and neurobiological perspectives

**DOI:** 10.3389/fnins.2013.00208

**Published:** 2013-11-07

**Authors:** Mike A. Miller, Alexander Thomé, Stephen L. Cowen

**Affiliations:** ^1^Department of Neuroscience, University of Arizona, Tucson, AZ, USA; ^2^Evelyn F. McKnight Brain Institute, University of Arizona, Tucson, AZ, USA; ^3^Department of Psychology, University of Arizona, Tucson, AZ, USA

**Keywords:** effort, risk, foraging, decision making, nucleus accumbens, dopamine, anterior cingulate cortex

## Abstract

The physical effort required to seek out and extract a resource is an important consideration for a foraging animal. A second consideration is the variability or risk associated with resource delivery. An intriguing observation from ethological studies is that animals shift their preference from stable to variable food sources under conditions of increased physical effort or falling energetic reserves. Although theoretical models for this effect exist, no exploration into its biological basis has been pursued. Recent advances in understanding the neural basis of effort- and risk-guided decision making suggest that opportunities exist for determining how effort influences risk preference. In this review, we describe the intersection between the neural systems involved in effort- and risk-guided decision making and outline two mechanisms by which effort-induced changes in dopamine release may increase the preference for variable rewards.

## Introduction

Foraging animals must often consider the variability or risk associated with resource delivery. For example, the preference for variable as opposed to stable sources of food can change dramatically based on a number of factors, such as an animal's current energetic state. In the case of food delivery, animals can express a preference for variable or “risky” food sources when the energetic demands of foraging are high (Caraco, 1981; Caraco et al., 1990). Juncos, for instance, prefer seed-bins with variable over fixed reinforcement schedules when ambient temperatures fall and metabolic demands rise (Caraco et al., 1990; but see Brito E Abreu and Kacelnik, 1999). Similarly, rats consuming high-calorie foods exhibit a reduced preference for variable schedules of reinforcement relative to groups of rats consuming low-calorie foods (Craft et al., 2011). Energetic expenditure due to physical effort can also enhance risk preference. For example, a study by Kirshenbaum et al. (2003) investigated the interaction between effort and risk in rats where effort was presented in the form of running in wheels with low (50 g) or high (120 g) spinning resistances. Risk was measured as the preference for variable or fixed schedules of reinforcement. The authors divided rats into high-effort and low-effort groups and observed that the high-effort group expressed a preference for variable reinforcement relative to the low-effort group. One explanation for this effect is the Daily Energy Budget (DEB) or Z-score rule (Stephens, 1981; Houston, 1991). According to this theory, when animals approach a critical energetic state (e.g., starvation), they choose to gamble by investing their remaining resources on a variable option that may yield a life-saving gain. This rule also states that organisms with adequate and stable energetic reserves will not make the gamble as they are better served by low-risk options that guarantee survival (For a thorough and critical review see Kacelnik and Bateson, 1996).

To our knowledge, no investigation into the neural basis for the influence of energetic state or physical effort on risk preference has been performed. Consequently, almost nothing is known regarding the neural basis for interactions between effort and risk. Opportunities do exist to explore this question given recent advances in the study of the neural basis of effort- and risk-guided decision making (Reviewed in Walton et al., 2006; Platt and Huettel, 2008; Salamone et al., 2012). These studies have identified a range of structures and neuromodulators involved in perceiving and setting the preference to exert effort or to pursue risky options. Surprisingly, the divergent networks involved in effort and risk intersect in a restricted set of cortical and subcortical structures; the most prominent being the anterior cingulate cortex (ACC), the basolateral amygdala (BLA), the nucleus accumbens (NAC), and the mesolimbic dopaminergic system. Given the considerable evidence for the role of these systems in setting the preference for both effort and risk, we believe that interactions between these structures underlie the capacity of physical effort to increase risk preference. To argue this point, we first review evidence that the neural processes involved in effort-guided (section Experimental Approaches for Studying Effort-Guided Behaviors, Cortical and Sub-Cortical Circuits Involved in Effort-Guided Behaviors and Dopamine and Effort-Guided Behavior) and risk-guided (section Neural Systems Involved in Setting The Preference for Risk) decision making intersect in these systems, and conclude with the discussion of a proposed neural mechanism by which effort increases the preference for risk, where risk preference is defined as a preference for variable over certain rewards.

## Experimental approaches for studying effort-guided behaviors

An underlying assumption of most investigations of effort-guided decision making is that effort is a cost to be minimized. This is a reasonable assumption given that many species balance energetic losses from physical effort against food intake in order to maximize net energetic gain (Stephens et al., 2007). Bautista et al. (2001) explored this issue in an experiment in which starlings choose between either walking (low effort) in search of low-yield rewards or flying (high effort) to reach larger rewards. Analysis of choice behavior in response to manipulations of reward magnitude and flight distance indicated that starlings optimized net energetic gain by shifting their preference between walking and flying. Similarly, food-restricted rats alter their preference for high-effort/high-reward and low-effort/low-reward options in ways that indicate that they treat effort as a cost (Reviewed in Walton et al., 2006; Salamone et al., 2012). In most neurobiological experiments, effort is manipulated using either lever-press behaviors coupled with variable or fixed-ratio schedules of reinforcement (Floresco et al., 2008; Walton et al., 2009) or barrier-climbing tasks where animals are required to select between paths on a maze that lead to combinations of effort (the presence of a 30–40 cm barrier) and reward (e.g., Salamone, 1994; Bardgett et al., 2009; Cowen et al., 2012). Importantly, results from a number of studies suggest that the observed aversion to effort reported in these studies is not due to an aversion to temporal delays produced by the time required to lever-press or jump over barriers (Rudebeck et al., 2006; Floresco et al., 2008; Walton et al., 2009). What follows is a review of the involvement of neural systems in effort-guided behaviors, with a focus on those systems that are also implicated in processing risk.

## Cortical and sub-cortical circuits involved in effort-guided behaviors

**Figure 1:**
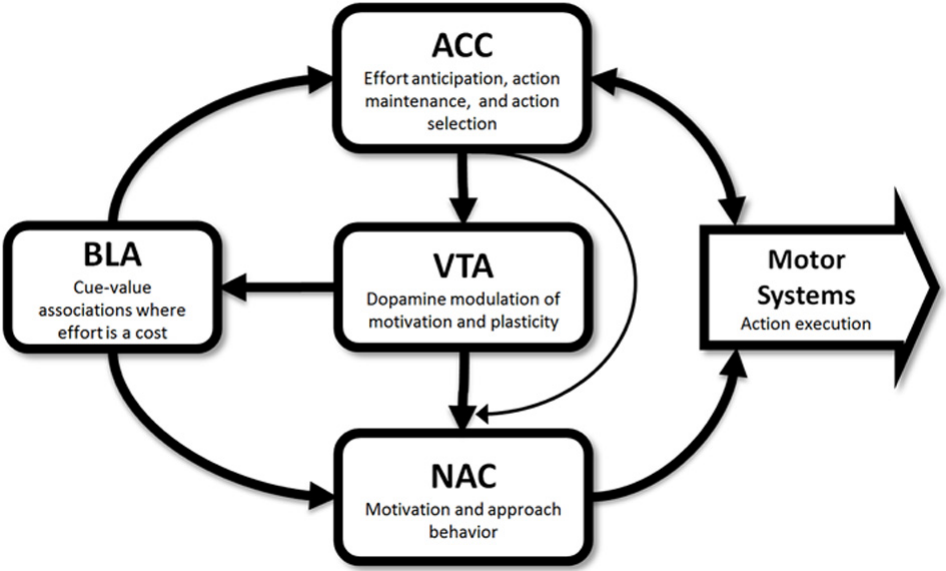
**Schematic of potential functions of neural systems within the effort network**. Results from studies in rodents and primates suggest the involvement of the anterior cingulate cortex (ACC), basolateral amygdala (BLA), nucleus accumbens (NAC), and dopamine neurons in the ventral tegmental area (VTA) in effort-guided decision making. Captions in each box indicate a proposed roles for each structure in effort-guided behavior and arrows indicate possible routes of information flow. These routes are based largely from the results of disconnection studies and anatomical studies performed in rodents.

Results from studies that have selectively targeted cortical and subcortical structures, either through lesions or through pharmacological manipulations, suggest the existence of a network involved in effort-guided decision making; with the principal nodes of this network being localized in the ACC, BLA, and NAC. For example, lesions or inactivation of the ACC result in animals avoiding previously preferred high-effort/high-reward options on the barrier-climbing task (Walton et al., 2003; Rudebeck et al., 2006; Floresco and Ghods-Sharifi, 2007). Notably, the placement of a second barrier on the formerly low-effort arm results in lesioned animals returning to the high-reward/high-effort option (Walton et al., 2002), suggesting that the observed shift in preference is not a consequence of an inability to climb the barrier or in capacity to recall reward values. This effect does not appear to extend to prefrontal regions ventral to the ACC as lesions of the prelimbic (Walton et al., 2003) and orbitofrontal cortex (Rudebeck et al., 2006) do not result in effort-avoidance. Similar patterns of effort-avoidance have been observed following disruption of subcortical regions such as the NAC and BLA. For example, effort avoidance has been reported in rats performing lever-press tasks following muscimol/baclofen inactivation of the NAC core (Ghods-Sharifi and Floresco, 2010) and on barrier-climbing tasks following the infusion of dopaminergic antagonists into the NAC (Salamone et al., 1994) or 6-OHDA dopamine depletion of the NAC (Mai et al., 2012). Similarly, muscimol/baclofen inactivation of the BLA (Ghods-Sharifi et al., 2009) on a lever-press task and excitotoxic lesions of the BLA in a barrier-climbing task (Ostrander et al., 2011) also produce effort-aversion in rats. Taken together, these data suggest the presence of a distributed cortical-subcortical network for effort processing. A schematic of this network along with possible modes of interaction is presented in Figure 1.

Although experiments such as those described above have identified individual structures involved in effort-related processing, little is known regarding the specific roles these structures play in effort-guided decision making or how interactions between structures influence behavior. Important steps in this direction have been made through the investigation of the behavioral consequences of the functional disconnection of structures within this “effort network.” For example, the functional disconnection of the BLA and ACC through the targeted inactivation of the BLA/ACC in contralateral hemispheres (disconnecting within-hemisphere communication) results in a degree of effort-avoidance that is similar to the degree observed following bilateral ACC or BLA lesions (Floresco and Ghods-Sharifi, 2007). In this study, lesions were in the most caudal region of the BLA, a region that does not receive significant input from the ACC (Sesack et al., 1989). This suggests that information could move from the BLA to the ACC during effort-guided decision making. Evidence for directional flow of information from the BLA to the ACC, along with an established role of the BLA in ascribing positive and negative affective values to stimuli and outcomes (Baxter and Murray, 2002) indicates that information about the effort-discounted value of rewards could be transferred from the BLA to the ACC during decision making (Floresco and Ghods-Sharifi, 2007). The ACC, given its role in integrating sensory and motor information in the service of action-selection (Shima and Tanji, 1998; Kennerley et al., 2009), may utilize information about outcome value from regions such as the BLA to bias action selection toward high-value options (Walton et al., 2006). This interpretation, however, must be tempered as it is possible that the ACC drives activity in the posterior BLA through indirect routes (e.g., through the anterior BLA). Future experiments involving simultaneous recording from the ACC and BLA during effort-guided behavior could further elucidate the question of the direction of information flow.

Interactions between the ACC and the NAC also appear to influence effort-guided behaviors. For example, the excitotoxic disconnection of the ACC and NAC core also results in effort-avoidance on a barrier-climbing task (Hauber and Sommer, 2009). There are multiple routes through which an ACC-NAC disconnection could disrupt inter-region communication. For example, projections from the ACC to NAC exist (Zahm and Brog, 1992; Voorn et al., 2004; Haber et al., 2006), although these are relatively weak (Gabbott et al., 2005). A second route is through the ventral tegmental area (VTA) as the ACC projects to the VTA (Oades and Halliday, 1987) and given that ACC stimulation is capable of activating VTA neurons (Gariano and Groves, 1988), and the VTA, in turn, projects to the NAC (Oades and Halliday, 1987). In contrast to the clear direct and indirect projections from the ACC to the NAC, there are few projections from the NAC to the ACC (Hoover and Vertes, 2007), suggesting that information flows from the ACC to the NAC (Hauber and Sommer, 2009). The specific involvement of the NAC in motivated approach behavior (Berridge and Robinson, 2003; Salamone et al., 2007) suggests that the NAC receives ACC output related to the chosen action, and utilizes this information to trigger or sustain an approach response.

In agreement with the proposed role of the ACC in action selection (Shima and Tanji, 1998), the preceding results suggests that the ACC integrates information about outcome values from regions such as the BLA in order to guide decision making. Support for a role of the ACC in action selection, however, is weakened by recent physiological observations in rodents that indicate that neural responses in the ACC to actions and to anticipated effort actually occur after action selection (Cowen et al., 2012). These observations are in agreement with an alternative view of ACC involvement in sustaining goal-directed responses across delays (Cowen et al., 2012). For example, Narayanan and Laubach (2006) demonstrated that ACC activity is required for sustaining a lever-press response in a time-estimation task. This conclusion is further supported by observations from primate studies that implicate the ACC in maintaining actions across trials (Kennerley et al., 2006; Hayden et al., 2011).

It must be cautioned that while these observations highlight similarities between primates and rodents, such comparisons are complicated by notable anatomical and functional differences between species (Preuss, 1995; Uylings, 2003; but see Vogt and Paxinos, 2012). For example, there is considerable evidence that the primate ACC can be divided into motor, cognitive, and affective components along the dorso-ventral/posterior-anterior axis (Barbas and De Olmos, 1990; Luppino et al., 1991; Bates and Goldman-Rakic, 1993; Barbas and Blatt, 1995; Picard and Strick, 1996), with most studies of effort-driven responses in primates targeting dorsal/motor regions (e.g., Kennerley et al., 2009). Although anatomical divisions along the anterior-posterior axis of the rodent ACC do appear to correspond generally to the divisions reported in primates (Vogt and Paxinos, 2012), few studies of behavioral/functional differences along this anterior-posterior axis in the rodent ACC have been performed. Interestingly, differences in motor sensitivity along the dorso-ventral axis of the rodent medial prefrontal cortex have been observed, with individual neurons in dorsal regions, including the ACC, exhibiting the greatest sensitivity to movement (Cowen and McNaughton, 2007).

## Dopamine and effort-guided behavior

Dopamine is traditionally viewed as a key modulator of reward-driven learning; however, the observation that dopamine modulates effort-guided behaviors suggests that this view requires elaboration (Wise, 2004; Salamone et al., 2012). For example, physical exercise in the form of swimming and running on a wheel or a treadmill and has been repeatedly associated with increases in dopamine concentration (Reviewed in Meeusen and De Meirleir, 1995). Such increases have been observed throughout the striatum (Freed and Yamamoto, 1985; Hattori et al., 1994; Meeusen et al., 1997), and a particularly strong relationship between dopamine concentration and running speed has been reported in the NAC (Freed and Yamamoto, 1985). In addition, rats receiving systemic administration of D1 (Bardgett et al., 2009) and D2 antagonists (Denk et al., 2005; Floresco et al., 2008; Bardgett et al., 2009; Salamone et al., 2012) shift their preference away from high-effort/high-reward options to low-effort/low-reward alternatives. Conversely, the application of the dopaminergic agonist D-amphetamine biases responses toward high-reward/high-effort options (Bardgett et al., 2009; but see Floresco et al., 2008).

The influence of dopamine on effort depends on dopamine's effect on specific structures within the effort-network. For instance, targeted 6-OHDA depletion of dopaminergic terminals or blockade of dopamine receptors within the NAC (Salamone, 1994; Cousins et al., 1996; Ishiwari et al., 2004) and the ACC (Schweimer and Hauber, 2006 but see Walton and Croxson, 2005) results in effort-avoidance during cost-benefit decision making. Furthermore, results from a microdialysis study of striatal dopamine during a random-ratio reinforcement task indicates that slow fluctuations in dopamine concentration (minutes) reflect an integration of factors such as motivational state (e.g., hunger) and the effort required to obtain rewards (Ostlund et al., 2011). Taken together, these data indicate that dopamine concentration within the ACC and the NAC influences the willingness of animals to exert physical effort in exchange for valued rewards. This function relates to recent theoretical proposals in which the level of tonic dopamine release modulates response vigor and response speed (Niv et al., 2005) as well as the estimation of long-term reinforcement rates (Niv et al., 2007; Ostlund et al., 2011).

Although there is clear evidence for a role of dopamine release in the NAC in effort-guided behaviors, virtually nothing is known regarding how this release is regulated. For example, the principal source of dopaminergic innervation of the NAC is the VTA, and it is unknown what systems drive VTA activity during effort-guided behaviors. The ACC could be involved given its capacity to trigger burst-firing in dopaminergic neurons (Gariano and Groves, 1988), and the previously described role of the ACC in sustaining motor actions (Narayanan et al., 2006; Cowen et al., 2012) would suggest, somewhat indirectly, ACC involvement in sustaining a dopaminergic response. An important path for future research is the exploration of interactions between systems within the effort network such as the ACC and BLA and the VTA.

Dopamine can modulate the activity of its targets across many time scales (Schultz, 2007), and the time scale most relevant for effort-guided behaviors has yet to be determined. For example, dopamine neurons can express tonic and phasic modes of firing activity (Grace, 1991), and these modes may play distinct roles in modulating effort-guided behaviors. Tonic activity is associated with slow release (seconds) of dopamine while phasic release is associated with brief (~300 ms) bursts of firing activity that produces large but temporary increases in dopamine concentration (Grace, 1991). There is some debate, however, regarding whether changes in extracellular dopamine concentration results from tonic or phasic release (Floresco et al., 2003; Owesson-White et al., 2012). Functionally, tonic patterns of firing activity of dopamine neurons have been associated with estimating the uncertainty of reward delivery in primates (Fiorillo et al., 2003); while phasic release is implicated in stimulus-driven orienting behaviors (Dommett et al., 2005) and associative plasticity (Hollerman and Schultz, 1998). Phasic and tonic patterns of release may also cooperate to regulate inter-region communication (Goto and Grace, 2005).

Little is known regarding the relative contribution of phasic and tonic release to effort-guided behaviors; however, three recent studies using fast scan cyclic voltammetry were unable to identify a clear effect of effort on phasic responses within the NAC (Day et al., 2010; Gan et al., 2010; Wanat et al., 2010). For example, two of these studies were unable to identify significant phasic response to effort-predictive cues in an instrumental task, but these studies did identify clear phasic responses to reward-predictive cues (Gan et al., 2010; Wanat et al., 2010). Furthermore, Day et al. (2010) reported an unanticipated reduction of phasic NAC dopamine release following increased effort. It is also unclear how short-duration phasic responses could exert the sustained effect on motivational state required to overcome costs such as physical effort (Niv et al., 2007). A possible way to accommodate this concern would be if phasic responses modulated the learning of associations between effort, stimuli, and outcomes during early stages of training as such learning could have lasting effects on behavior. Future work in this area may yield important results given considerable evidence for a role of phasic dopamine in learning (Schultz et al., 1997; Waelti et al., 2001). Indeed, such investigations may explain the difficult to resolve issue of how effort, under the right conditions, can enhance the reinforcing value of effort-associated cues, actions, and primary rewards (Eisenberger, 1992; Zentall, 2010; Johnson and Gallagher, 2011).

The variable effects of dopamine on effort-guided behaviors and the different timescales at which dopamine influences neural activity present significant challenges to researchers hoping to create a unifying theory of function. These challenges are compounded by experimental results that suggest that the involvement of dopamine and the NAC in effort-guided behaviors depends on the specific form of physical effort being employed. For example, 6-OHDA depletion of NAC dopamine significantly impairs effort-guided behavior when effort is assessed using fixed-ratio schedules; however, the same dopaminergic manipulation has no effect when effort is in the form of a weighted lever (Ishiwari et al., 2004). One interpretation of these different results is that NAC dopamine is required for sustaining effort over time. This interpretation, however, does not fit well with results from T-maze barrier climbing tasks in which the time required for expending effort within a trial is quite brief. Instead, dopaminergic manipulations may be most effective when sequences of effortful motor acts must be maintained. For example, the degree of motor control required to press a weighted lever a single time is probably similar to the control required for a single press of an unweighted lever. In contrast, the control required to execute a single lever-press is probably considerably less than what is required to sustain a repeating sequence of presses or, in the case of barrier-climbing, in executing a complex jumping procedure. This idea may relate to a formulation of decision-related costs proposed by Shenhav et al. (2013) in which cost is the degree to which control mechanisms must be engaged during a behavior. The authors go further to suggest that the dorsal ACC allocates control to a given mental or physical operation based on the estimated value of the outcome. This value signal used in this allocation process is suggested to arrive from regions such as the amygdala, insula, and dopaminergic system (Shenhav et al., 2013).

## Neural systems involved in setting the preference for risk

Studies in humans, non-human primates, and rodents have identified diverse cortical and subcortical structures involved in setting the preference or aversion to risk. In human studies, the Iowa Gambling Task has become an established task for investigating the neural basis for risk-guided decision making (Bechara et al., 1994). In this task, subjects choose between four decks of cards with the decks having different levels of probabilistic gains and losses and different long-term returns. Risk-preference is measured as a preference for suboptimal high-risk/high-reward decks over optimal low-risk alternatives. A preference for high-risk options has been observed in patients with damage to the ventromedial prefrontal cortex and amygdala (Bechara et al., 1994, 1999). Furthermore, BOLD activation of the medial frontal gyrus, lateral orbitofrontal cortex, and insula increases when subjects choose suboptimal high-risk options (Lawrence et al., 2009).

A criticism of the use of the Iowa Gambling Task for assessing risk-preference is that the task places multiple cognitive demands upon subjects that are unrelated to risk. This is problematic as a preference for risky alternatives or neural responses related to risk preference could, in this task, result from a reduced capacity to shift attention, to perform behavioral reversals, to process losses, or to store items in working memory (Maia and McClelland, 2004; Dunn et al., 2006). To address this issue, some fMRI studies in humans and most studies in non-human primates and rodents use paradigms that more directly assess the preference or aversion to variability in reward delivery. These studies have implicated a large number of structures in risk-guided decision making such as the lateral frontal cortex (Tobler et al., 2009), medial prefrontal cortex (St. Onge and Floresco, 2010), insula (Preuschoff et al., 2008; Rudorf et al., 2012), posterior parietal cortex (Huettel et al., 2006), ACC (Christopoulos et al., 2009; Kennerley et al., 2009), posterior cingulate cortex (McCoy and Platt, 2005), VTA (Fiorillo et al., 2003), putamen (Preuschoff et al., 2008), and ventral striatum (Cardinal and Howes, 2005; Preuschoff et al., 2006; Stopper et al., 2013).

Connecting results from human studies to results from studies using non-human primates and rodents is a significant challenge given the wide range of structures identified within and across species. Even so, there are points of intersection. Interestingly, many of these points of intersection include elements of the effort network, such as the ACC, NAC, BLA, and dopamine (Amiez et al., 2006; Behrens et al., 2007; Pais-Vieira et al., 2007; Platt and Huettel, 2008; Preuschoff et al., 2008; Choi and Kim, 2010; Fitzgerald et al., 2010; Rivalan et al., 2011; Schultz et al., 2011; Zeeb and Winstanley, 2011). The following sections review the involvement of these systems in risk-guided behaviors.

### Risk and the anterior cingulate cortex

Multiple lines of evidence suggest a role of the primate ACC in evaluating risk and in altering risk preference. In non-human primates, the spiking activity of subsets of ACC neurons changes monotonically with the probability that a cue predicts reward (Kennerley et al., 2009). In humans, BOLD activity in the ACC increases prior to decisions involving risk (Labudda et al., 2008; Weber and Huettel, 2008; Lawrence et al., 2009; Smith et al., 2009). Furthermore, the combination of BOLD activation of the ACC and striatum predicts risk-preference on subsequent trials (Christopoulos et al., 2009), suggesting that ACC/striatal activation sets a baseline preference for risk. The ACC may specialize in monitoring a form of risk known as volatility or “uncertain uncertainty,” a form of uncertainty in which distributions and means of outcome delivery are unknown (Rushworth and Behrens, 2008). BOLD activity in the ACC scales with the amount of information an outcome provides about the underlying outcome distribution (Behrens et al., 2007). For a foraging animal, adapting to volatility is important as it determines how much weight to place on the history of reward delivery, with high volatility indicating that more attention should be paid to recent history. Results from lesion studies of the primate ACC suggest that ACC plays an important role in tracking reward history (Kennerley et al., 2006) and so ACC responses to volatility would support this function.

When compared to results from primates, results from studies in rodents have not produced consistent evidence for a role of the ACC in risk-guided behaviors. Although inactivation (St. Onge and Floresco, 2010), lesions (Rivalan et al., 2011), or dopaminergic blockade (St. Onge et al., 2011) of the medial prefrontal cortex does alter risk-preference, studies that have specifically targeted the rodent ACC have not reported alterations in risk preference in two tests of risk preference (St. Onge and Floresco, 2010; Rivalan et al., 2011).

### Risk, dopamine, and the nucleus accumbens

Dopamine plays a central albeit complex role in modulating the preference for risk. For example, systemic administration of dopamine D1 and D2 antagonists in rats results in the avoidance of high-risk/high-reward options in a 2-lever risk-discounting task (St. Onge and Floresco, 2009) while amphetamine or D1 or D2 agonists increased the preference for high-risk/high-reward options in the same task (St. Onge and Floresco, 2009). Conversely, a study that combined the risk of reward with the risk of punishment (foot shock) reported that systemic administration of D2 agonists and amphetamine reduced risk taking for suboptimal large-reward/punishment options (Simon et al., 2011). Similarly, another study reported that the systemic application of D2 antagonists resulted in the avoidance of high-risk/reward options on a rodent version of the Iowa Gambling Task that also incorporated punishment in the form of time-outs (Zeeb et al., 2009). The specific effects of systemic D2 antagonism can be difficult to interpret given that D2-like receptors are located on presynaptic dopaminergic afferents and on the postsynaptic terminals of their cortical and striatal targets (Palij et al., 1990; Timmerman et al., 1990; Santiago and Westerink, 1991). Taken together, these results do indicate that dopamine plays a clear but complex role in risk-guided behaviors, and the specific role may depend on the task, the presence or absence of punishment, and the receptor type that is targeted.

Results from single unit-studies of dopaminergic neurons in the primate VTA also suggest a role of dopamine in evaluating risk. For example, the activity of dopaminergic neurons follows the probability of receiving a future reward with the activity of these neurons peaking when the capacity to predict a reward is lowest (Fiorillo et al., 2003; Tobler et al., 2005). Similarly, imaging studies of the midbrain in humans show increased BOLD activation under conditions of outcome uncertainty (Aron et al., 2004). The effects of dopaminergic manipulations in humans also support a role for dopamine in shaping risk preference. For example, patients undergoing dopamine agonist therapy for the treatment of Parkinson's disease and restless leg syndrome exhibit a four-fold increase in gambling-related problems (Dodd et al., 2005; Dang et al., 2011), while low doses of amphetamine increase self-reports of the desire to gamble in problem gamblers (Zack and Poulos, 2004). Interestingly, the application of antagonists that targeted only D2 receptors increased the perceived reward of a slot-machine gambling episode (Zack and Poulos, 2007). Finally, changes in striatal and cortical dopamine receptor populations and dopamine transporters (DAT) over an organism's development correspond with age-associated shifts in risk preference (Wahlstrom et al., 2010).

As in effort-guided decision making, the influence of dopamine on risk preference may be through dopamine's regulation of the NAC. For example, inactivation or lesions of the NAC in rodents reduces the preference for large, uncertain rewards (Cardinal and Howes, 2005; Stopper et al., 2013). Furthermore, infusion of D1 antagonists into the NAC of rats decreases the preference for larger, uncertain rewards on a probability discounting task. In contrast, infusion of a D1 or D2/D3 agonists into the NAC **i**ncreases risk preference when reward probability is high (Norbury et al., 2013; Stopper et al., 2013; however, see Mai and Hauber, 2012). Infusion of D1 agonists into the NAC may also “optimize” decision making in treated animals as one study reported that treated rats were more likely to choose the large and uncertain reward when reward probability was high, but were more likely to avoid this option when probabilities were low (Stopper et al., 2013). Further, background dopamine levels appear to peak on trials during which reward delivery is uncertain (St. Onge et al., 2012a). Similarly, one study reported that BOLD responses in the human NAC were largest under conditions of maximal uncertainty (Preuschoff et al., 2006). In another study, NAC BOLD activation increased before subjects made risky choices in a task involving decisions between high (stocks) or low (bonds) risk investments (Kuhnen and Knutson, 2005). These patterns of activation mirror patterns observed in the firing activities of dopaminergic neurons of the VTA of non-human primates (Fiorillo et al., 2003; Tobler et al., 2005).

Taken together, these results suggest that increased NAC dopamine enhances the tolerance of risk while reductions in NAC dopamine reduces risk preference. A similar pattern was observed for effort, with enhanced NAC dopamine resulting in an increased willingness to work for rewards and reduced dopamine resulting in effort avoidance. The following section presents possible mechanisms by which these similar patterns contribute to the influence of effort on risk preference.

### Risk and the basolateral amygdala

The BLA is also associated with the assessment of risk, although fewer studies have targeted the BLA relative to the regions discussed previously. For example, results from a study by Ghods-Sharifi et al. (2009) suggest that inactivation of the BLA leads to risk-averse behavior. In this study, animals chose between a lever that delivered a small, certain reward and a lever that delivered a large reward with decreasing reinforcement probabilities that shifted from certain (reward on 100% of trials) to risky (reward on 12.5% of trials). Rats with bilateral inactivation of the BLA preferred the low-risk lever when compared to the saline control. In the same study, the authors investigated effort discounting using a ratio schedule of reinforcement and reported that BLA lesions also resulted in effort aversion (Ghods-Sharifi et al., 2009). Taken together, this suggests a general role for the BLA in overcoming costs associated with both effort and risk. However, observations from a recent disconnection study performed by the same group suggest that the role of the BLA and its interaction with other structures is nuanced. In this study, the authors disconnected the BLA and mPFC and the BLA and the NAC in a similar risk-discounting task (St. Onge et al., 2012b). The authors observed that BLA-mPFC disconnection resulted in increased risk preference while BLA-NAC disconnection resulted in risk aversion. The study further determined that it was the mPFC to BLA and not the BLA to mPFC connection that was involved in enhancing risk-preference. This finding suggests that the mPFC input to the BLA could serve to blunt risk-seeking so that animals can optimize foraging behavior over time.

## How does effort alter risk preference?

Increased metabolic demands and expenditure of physical effort can enhance the preference for variable rewards (Caraco, 1980, 1981; Caraco et al., 1990; Kirshenbaum et al., 2003). It has been proposed that a shift in preference for variable rewards is an adaptive behavior that biases animals to gamble on potentially large and life-sustaining gains (Stephens, 1981). The neural mechanisms underlying the capacity of effort to alter risk preference are unknown. Broadly, there are at least two routes by which effort could influence risk. First, the notable intersection between the neural systems involved in effort and risk-guided behaviors (e.g., the NAC, ACC, and BLA) and the capacity neurons within some of these structures to respond to both effort and risk (e.g., Kennerley et al., 2009) suggests that activity in these structures that results from anticipated or experienced effort could alter and consequently bias the processes within these structures involved in evaluating risk. Alternatively, it is possible that neural structures that are not typically associated with effort or risk could become engaged only when environmental contingencies require the integration both of these considerations (Burke et al., 2013). For example, Burke et al. (2013) observed that significant BOLD activation of the frontal pole was only observed when combinations of effort and risk were being considered. The authors of this study did not investigate the neural basis for the capacity of effort to alter risk-preference, and, to our knowledge, no direct investigation of this question has been performed. It is therefore hoped that the following and admittedly speculative proposals will motivate future experiments to fill this gap in understanding.

### Effort-triggered increases in NAC dopamine concentration enhances risk preference

Dopamine is an important modulator of both effort- and risk-guided behaviors. Here we propose that the release of dopamine in the NAC in response to physical effort enhances risk preference. This idea is supported by the following observations. First, dopamine concentrations within the NAC increase with physical exercise (Freed and Yamamoto, 1985). Furthermore, increases in dopamine concentration are associated with increased risk preference in humans (Zack and Poulos, 2004; Dodd et al., 2005; Dang et al., 2011) and rodents (St. Onge and Floresco, 2009; Stopper et al., 2013). Finally, stimulation of dopamine D1 receptors within the NAC enhances risk preference (Stopper et al., 2013; however, see Mai and Hauber, 2012). Consequently, we propose that effort-induced increases in NAC dopamine concentration drives risk preference by enhancing dopamine release in the NAC.

What remains to be specified is the mechanism by which NAC dopamine concentration is regulated during effort-guided behaviors. In this regard, the ACC may play a central role given abundant evidence for ACC involvement in effort-guided behaviors (Reviewed in section Experimental Approaches for Studying Effort-Guided Behaviors, Cortical and Sub-Cortical Circuits Involved in Effort-Guided Behaviors and Dopamine and Effort-Guided Behavior and see Figure 1). Furthermore, activation of the ACC is capable of driving the responses of dopaminergic neurons in the VTA (Gariano and Groves, 1988), the principal source of NAC dopamine. Activity within the dorsomedial prefrontal cortex, including the ACC, is also capable of sustaining motor activity during delay intervals (Narayanan and Laubach, 2006; Narayanan et al., 2006) suggesting that ACC neurons could sustain dopaminergic responses and enhance dopaminergic tone in the NAC during effortful behaviors. The increase in NAC dopaminergic tone could, as a secondary effect, alter risk-guided behaviors.

The capacity of the ACC to sustain responses over delays may be particularly important for maintaining effortful behaviors as such behaviors often require maintaining motor responses over seconds and minutes. Consequently, understanding the timescale of dopamine release in the NAC during effortful behavior may be critical for understanding its role in modulating risk-preference. As discussed in section Dopamine and Effort-Guided Behavior, dopamine release can occur on multiple timescales, and the timescale most relevant for effort- and risk-guided behaviors has yet to be determined, although evidence is building that phasic patterns of dopamine release in the NAC are not critical for effort-guided behaviors (Gan et al., 2010; Wanat et al., 2010). Instead, gradual shifts in dopamine release may modulate effort-guided decision making, facilitate the maintenance of effortful responses, and sustain a preference for risk. Increases in tonic firing of VTA neurons correlates with anticipated risk (Fiorillo et al., 2003), suggesting that effort-induced increases in dopamine may alter risk-preference.

Interestingly, a recent study has identified within-trial increases in striatal dopamine concentration that ramps up as rats approach goal locations on a T-maze (Howe et al., 2013). These increases appear to be independent of phasic dopamine release and occur at a faster time scale than typically reported for changes in background dopamine concentration. The ACC could potentially drive this ramping response given the capacity of medial prefrontal neurons to sustain firing activity in afferent targets (Narayanan and Laubach, 2006; Narayanan et al., 2006). As suggested by Howe et al. (2013), the ramping response may be involved in setting the ongoing level of motivation. Determining the level of motivation could require the integration of factors such as effort, risk, and expected reward.

### Effort-triggered increases in dopamine enhances learning under conditions of uncertainty

The preceding account suggests that effort-induced changes in NAC dopamine enhance risk-preference and provides the motivational signal required for animals to sustain effortful behaviors. An alternative mechanism by which effort could influence risk is through dopamine's impact on stimulus-reward learning. Dopamine has an established role in facilitating such learning (Schultz et al., 1997; Day et al., 2007; Flagel et al., 2011; Steinberg et al., 2013), and both tonic and phasic patterns of dopamine release appear to enhance plasticity at hippocampal-NAC synapses (Goto and Grace, 2005). It is therefore possible that increases in NAC dopamine from both effort (Freed and Yamamoto, 1985) and risk (Fiorillo et al., 2003; St. Onge et al., 2012a) could produce an additive effect on plasticity, and, as a result, an additive effect on stimulus-reward learning under conditions that combine risk and effort. Anatomical connectivity between the VTA and the amygdala (Oades and Halliday, 1987) and the capacity of dopamine to gate plasticity within the amygdala (Bissière et al., 2003) suggest that enhancements in plasticity could extend to other limbic structures.

An interesting feature of this idea is that it may account for the paradoxical capacity of effort to enhance the reinforcing properties of cues and primary rewards. Pigeons, for example, prefer colored lights, spatial locations, and primary rewards associated with high effort (Clement et al., 2000; Friedrich and Zentall, 2004; Singer et al., 2007). Similar observations have been reported in starlings (Kacelnik and Marsh, 2002), mice (Johnson and Gallagher, 2011), and humans (Zentall, 2010). Indeed, mice can express a preference for low- over high-calorie foods when low-calorie foods are paired with high effort (Johnson and Gallagher, 2011). If effort-triggered dopamine release enhances stimulus-reward learning in, for example, the amygdala (Bissière et al., 2003), or striatum (Day et al., 2007; Cerovic et al., 2013), then such learning could result in a preference for rewards and reward-predictive cues learned under conditions of effort.

## Conclusion

The recent trend of using ethologically motivated experimental design to connect field observations with observations from neuroscience (Glimcher, 2002; Bateson, 2003; Stephens, 2008; McNamara and Houston, 2009) has begun to produce interesting results (e.g., Pearson et al., 2009; Hayden et al., 2011; Kolling et al., 2012). One important insight from foraging research is that animals are particularly attentive to the variability of reward delivery, even when average amounts of rewards are held constant (Stephens and Krebs, 1987; Kacelnik and Bateson, 1996). A second interesting observation is that the preference for such variability can be modulated by energetic state (Caraco, 1981) and physical effort (Kirshenbaum et al., 2003). Although there is compelling behavioral evidence for interactions between effort and risk, neuroscientific investigations of these interactions have not been performed. Given the surprising degree of overlap between networks involved in effort- and risk-guided behaviors, there is good reason to believe that the neural systems involved in evaluating, processing, and setting the preference for effort and risk interact. Consequently, it is hoped that this review will stimulate interest in the risk-effort connection and encourage new experiments that investigate the nuanced role that physical effort plays in foraging and decision making.

### Conflict of interest statement

The authors declare that the research was conducted in the absence of any commercial or financial relationships that could be construed as a potential conflict of interest.
